# High-Precision Multi-Axis Robotic Printing: Optimized Workflow for Complex Tissue Creation

**DOI:** 10.3390/bioengineering12090949

**Published:** 2025-08-31

**Authors:** Erfan Shojaei Barjuei, Joonhwan Shin, Keekyoung Kim, Jihyun Lee

**Affiliations:** 1 Department of Mechanical and Manufacturing Engineering, University of Calgary, Calgary, AB T2N 1N4, Canada; erfan.shojaeibarjuei@ucalgary.ca (E.S.B.); joonhwan.shin@ucalgary.ca (J.S.); keekyoung.kim@ucalgary.ca (K.K.); 2 Department of Biomedical Engineering, University of Calgary, Calgary, AB T2N 1N4, Canada

**Keywords:** bioprinting process, robotic platform, 3D bioprinting, embedded bioprinting

## Abstract

Three-dimensional bioprinting holds great promise for tissue engineering, but struggles with fabricating complex curved geometries such as vascular networks. Though precise, traditional Cartesian bioprinters are constrained by linear layer-by-layer deposition along fixed axes, resulting in limitations such as the stair-step effect. Multi-axis robotic bioprinting addresses these challenges by allowing dynamic nozzle orientation and motion along curvilinear paths, enabling conformal printing on anatomically relevant surfaces. Although robotic arms offer lower mechanical precision than CNC stages, accuracy can be enhanced through methods such as vision-based toolpath correction. This study presents a modular multi-axis robotic embedded bioprinting platform that integrates a six-degrees-of-freedom robotic arm, a pneumatic extrusion system, and a viscoplastic support bath. A streamlined workflow combines CAD modeling, CAM slicing, robotic simulation, and automated execution for efficient fabrication. Two case studies validate the system’s ability to print freeform surfaces and vascular-inspired tubular constructs with high fidelity. The results highlight the platform’s versatility and potential for complex tissue fabrication and future in situ bioprinting applications.

## 1. Introduction

Bioprinting technologies have evolved rapidly in recent years, offering multiple approaches for fabricating complex biological structures. The three most widely used bioprinting modalities are: (1) extrusion-based printing, which deposits continuous filaments of bioink through pneumatic or mechanical forces; (2) droplet-based methods such as inkjet or microvalve bioprinting, which deposit discrete volumes of material; and (3) light-assisted techniques, including micro-stereolithography (μSL), two-photon polymerization (2PP), and holographic bioprinting [1,2]. Light-based methods are particularly well suited for high-resolution fabrication, and are capable of generating highly complex 3D geometries with cell-level precision. For instance, 2PP can achieve feature resolutions below 200 nm [3], while μSL enables voxel sizes of 1–10 μm [4]. Moreover, sacrificial templating strategies have enabled the construction of intricate vascular networks [5], further expanding the potential of non-extrusion approaches.

Despite their precision, light-assisted methods often rely on photocrosslinkable materials and involve complex optical setups, which can limit their use with certain cell types or in surgical environments. In contrast, extrusion-based bioprinting remains the most commonly used technique due to its simplicity, broad material compatibility, and suitability for printing high-viscosity bioinks and cell-laden hydrogels. Extrusion bioprinters typically achieve feature resolutions in the range of 100–500 μm, with surface roughness between 50–150 μm depending on bioink properties and nozzle diameter [6,7]. These characteristics make extrusion well suited for integrating with robotic platforms, particularly in environments requiring adaptability to irregular surfaces or extended reachability.

However, traditional extrusion-based bioprinters often rely on layer-by-layer Cartesian mechanisms, operating along linear directions in the *x*, *y*, and *z* axes [8]. While z-height can be adjusted within a layer to enable non-planar constructs [9], most commercial systems remain constrained to planar slicing and stacked deposition due to limitations in their motion and software toolchains. As a result, they exhibit reduced geometric fidelity and an inability to print overhangs exceeding 45–60° without support structures [10]. Furthermore, their positional accuracy is often limited to ±100 μm, and smallest reliably printable features remain above 150–200 μm [11].

In contrast, robotic systems particularly those using 6-DOF arms—introduce enhanced directional flexibility. These systems allow real-time adjustment of nozzle orientation, enabling deposition on arbitrarily curved surfaces without introducing stair-step artifacts [12]. While robotic arms typically offer lower mechanical precision (e.g., UR5e repeatability: ±30 μm) than CNC-grade Cartesian systems (e.g., ±10 μm) [13], their advantage lies in expanded workspace (up to 850 mm radius for UR5e) and ability to print at varying angles and undercuts without supports [14]. For example, robotic bioprinting platforms have successfully maintained nozzle–surface normality within ±5° during deposition, which improves filament width consistency and reduces variability to below 10% across complex surfaces [15,16].

Moreover, robotic platforms can perform *conformal printing* in which the nozzle follows the topography of the target surface, enabling printing on biologically relevant shapes. This is particularly important for anatomical constructs such as tracheal or vascular grafts. Compared to three-axis systems, which struggle with such geometries, robotic systems reduce the need for sacrificial support by over 60% and improve print-to-CAD fidelity by 15–25% depending on surface curvature [17,18].

Despite these advantages, multi-axis robotic bioprinting introduces challenges in toolpath planning, control integration, and hardware–software interfacing. Standard STL-based slicers cannot natively support omnidirectional printing paths, and real-time orientation planning requires advanced surface-following algorithms [18]. Nonetheless, recent studies have shown that with custom slicing and AI-assisted planning, robotic systems can fabricate non-planar structures in half the time needed by traditional approaches while preserving structural integrity [19].

To further expand the range of printable structures, embedded bioprinting has gained attention. In this method, soft or low-viscosity bioinks are deposited into a viscoplastic support bath [20,21]. The bath behaves as a Bingham plastic [22], exhibiting solid-like behavior under low shear stress and flowing under higher shear stress [23,24]. This allows the nozzle to move freely while the structure is supported, enabling fabrication of unsupported features such as overhangs, bridges, and hollow channels [25,26].

In this study, we present a modular multi-axis robotic embedded bioprinting platform that integrates a 6-DOF robotic arm and a pneumatic extrusion system to fabricate complex tissue structures within a support bath. The system operates within a streamlined digital process chain tailored for robotic path planning and multi-directional control. While the current implementation focuses on embedded bioprinting, the architecture is inherently adaptable. The same platform can be reconfigured for in situ bioprinting by removing the support bath and employing real-time surface mapping. With a large reachable workspace, programmable end-effector orientation, and capability for continuous toolpath adjustment, this platform provides a versatile solution for both preclinical tissue engineering and intraoperative bioprinting applications.

To summarize the structure of this paper: Section 2 describes the materials used and the development of the multi-axis robotic bioprinting platform, including the hardware setup, control system, and optimization of printing parameters; Section 3 presents two case studies that validate the platform’s capabilities in fabricating complex and biologically relevant geometries; Section 4 provides a detailed analysis of the rheological properties, printing results, and comparative performance assessments; finally, Section 5 concludes the study and outlines future directions for advancing the platform toward clinical and translational bioprinting applications.

## 2. Materials and Methods

### 2.1. Preparation of Bioinks

In this study, a Gelatin methacryloyl (GelMA)–alginate precursor solution was used as the bioink material. GelMA-based hydrogels are widely recognized for their biocompatibility, tunable physical properties, and biofunctionalization [27]. To enhance viscosity and mechanical strength while preserving cell viability, sodium alginate was incorporated into GelMA, resulting in a composite with improved structural integrity [28]. Due to the distinct gelation mechanisms of GelMA and sodium alginate, the bioink undergoes a two-step gelation process to form GelMA-alginate hydrogels [29]. First, GelMA is crosslinked under a 405 nm light source. Then, sodium alginate is crosslinked using a 1 mM calcium chloride (CaCl_2_) solution. This sequential crosslinking forms individual polymer networks, the interpenetration of which results in the formation of GelMA–alginate hydrogels [30].

The GelMA was synthesized following the protocol outlined by Kumar et al. [31], with some modifications. The process began with dissolving 5 g of powdered gelatin derived from porcine skin (Type A, Bloom strength 300, Sigma-Aldrich, St. Louis, MO, USA) in 50 mL of deionized water at 50 °C to ensure complete dissolution. Subsequently, 10 mL of glycidyl methacrylate (Sigma-Aldrich) was added dropwise to maintain a stable reaction environment. The mixture was stirred continuously at 50 °C for 12 h to allow for a complete reaction. Afterward, the solution underwent dialysis for three days at room temperature using reverse-osmosis water, which was replaced twice daily to remove any unreacted glycidyl methacrylate and ensure the purity of the GelMA. The purified samples were then lyophilized to remove all water content, yielding the final GelMA product. To preserve its integrity, the GelMA was stored at −20 °C until needed.

For this study, the GelMA concentration was 10% *w*/*v* and the sodium alginate concentration was 1% *w*/*v*. These concentrations were chosen based on the findings of Krishnamoorthy et al. [30], which demonstrated their favorable physical properties for 3D bioprinting. To prepare the GelMA–alginate precursor solution, the lyophilized GelMA and sodium alginate powder were dissolved in deionized water, followed by the addition of 10% *v*/*v* of an Eosin Y (EY)-based photoinitiator stock solution. This photoinitiator stock solution consisted of 2 mM EY and 20% *w*/*v* triethanolamine (TEOA) dissolved in deionized water. The 10% *v*/*v* refers to the volume of this stock solution relative to the total volume of the GelMA–alginate precursor solution.

Before use, the GelMA–alginate precursor solution was left at room temperature (22 °C) for 30 min to ensure complete dissolution and homogeneity.

### 2.2. Preparation of the Suspension Bath

Carbopol suspension (0.4% *w*/*v*) was chosen as the suspension bath for this study due to its cell-friendly nature, stability, tunability, and viscoplastic properties [32]. Compared to other commonly used support media such as gelatin slurry and alginate microparticles, Carbopol gel allows for a more efficient preparation process, requiring fewer steps and less time [33]. Additionally, it enables the use of lower alginate concentrations in gelatin–alginate composite hydrogels, which is advantageous since higher alginate concentrations can reduce printing accuracy by increasing viscosity and limiting flow [34].

The selected Carbopol concentration was based on the findings of Ning et al. [23], who demonstrated that this concentration optimally balances mechanical support and print flow while maintaining high printing fidelity.

Preparing the Carbopol suspension (0.4% *w*/*v*) began by dissolving Carbomer 940 powder in deionized water at the specified concentration and allowing it to rest for 24 h. The solution was then divided into 35 mL portions, with each portion placed into a 50 mL centrifuge tube. To adjust the pH, 50 μL of a 10% NaOH solution was added to each batch.

Next, the Carbopol medium was degassed by centrifuging it at 1500 rpm for 10 min. Degassing is a crucial step, as it removes air bubbles that could compromise the structural integrity of the bioprinted scaffolds. Degassing was performed in centrifuge tubes to ensure uniform and efficient bubble removal prior to transfer. Degassing directly inside the printing vat was avoided due to volume limitations and uneven degassing efficiency. While additional bubbles can be eliminated by storing the medium at 4 °C, centrifugation significantly reduces larger air pockets, enhancing print consistency.

After degassing, the Carbopol medium was carefully transferred into a custom-made container measuring 20 cm × 20 cm × 10 cm, ensuring that no new air bubbles were introduced during the transfer. In the event that any air bubbles formed, larger ones were manually extracted using a syringe, while smaller bubbles were removed by storing the Carbopol medium at 4 °C for 24 h.

Finally, the prepared Carbopol gels were stored at 4 °C until needed. Post-printing, the Carbopol gel facilitates the quick and non-destructive release of printed constructs by washing with phosphate-buffered saline (PBS) [23].

### 2.3. Development of the Multi-Axis Robotic Bioprinting Platform

The main components of the robotic bioprinter include a robotic arm, a pneumatic extrusion system, a digital camera, and the supervisor’s computer. A desktop robotic arm (the UR5e model manufactured by Universal Robots) is mounted on a table to ensure precise positioning of the nozzle. This robotic arm has a repeatability of ±0.03 mm and a working radius of 850 mm.

A pneumatic dispenser (model 983A, Musashi Engineering, Tokyo, Japan) is integrated within the robotic arm’s control box to dispense bioink. It is connected to an air supply via a pressure gauge through a flexible tube. Additionally, a syringe is mounted on the robotic arm’s end-effector using a 3D-printed holder and is linked to the pneumatic dispenser by another flexible tube. A digital pressure gauge is installed in the tubing between the dispenser and the syringe to actively regulate pressure. A 23-gauge dispensing needle serves as the nozzle.

A digital camera (model acA3088-57µm, Basler, Ahrensburg, Germany) equipped with a lens rated for 10 MP resolution (model HIKROBOT MVL-HF2524M-10MP, HIKROBOT, Hangzhou, China) is also mounted on the end-effector of the robotic arm, utilizing the same 3D-printed mount for print analysis. The digital camera has a resolution of 6 MP.

**Figure 1:**
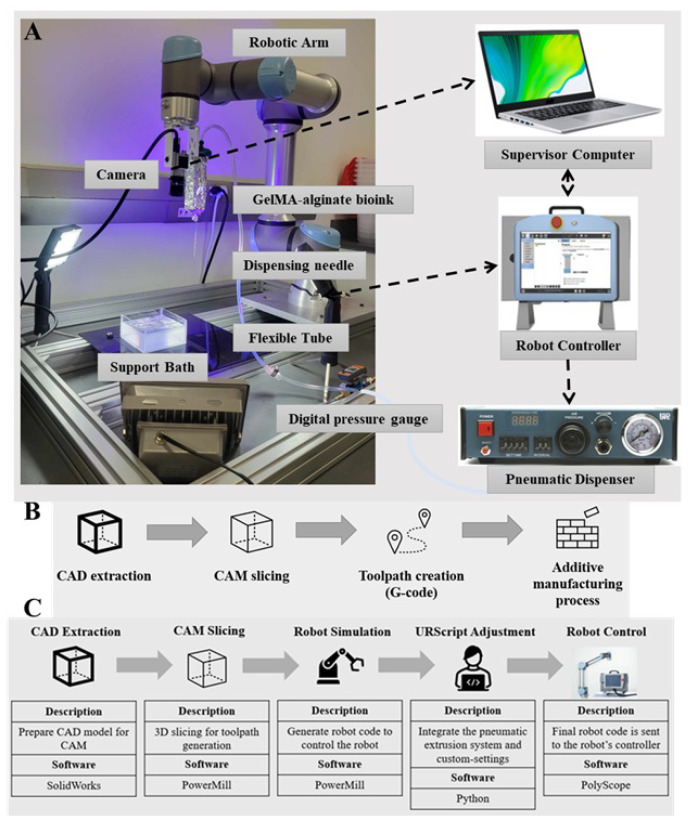
The developed multi-axis robotic bioprinting platform: (**A**) the main components of the platform, (**B**) conventional process chain of additive manufacturing, and (**C**) overview of the process chain for the developed robotic bioprinter.

The supervisor’s computer is responsible for generating, editing, and modifying files that are sent to the robot controller for the bioprinting process. Additionally, it receives data from the digital camera. This computer is connected to the robot controller via ethernet connection and to the digital camera through a USB 3.0 connection. Figure 1A illustrates the multi-axis robotic bioprinting platform along with all its components.

### 2.4. Integrated Control System for Robotic Bioprinting

The bioprinting platform was controlled using URScript, the proprietary programming language of Universal Robots, operating primarily at the script level to ensure precise nozzle positioning and consistent bioink extrusion. The robotic arm executed linear toolspace trajectories to maintain a constant linear velocity of the nozzle tip along the deposition path. To achieve conformal deposition on complex surfaces, the system dynamically computes the surface normal at each toolpath point using the local geometry extracted from the CAD model. The orientation of the 6-DOF robotic arm’s end-effector is continuously adjusted in real time to align with the calculated surface normal, minimizing deviations and ensuring uniform filament width and layer conformity. Orientation errors were measured along representative freeform paths and remained within ±5°, confirming effective surface-normal tracking even on steep or curved geometries (Table 1).

Computer-aided manufacturing (CAM) software was used to generate the toolpath by defining Cartesian coordinates and orientation vectors, ensuring accurate motion control. A custom Python 3.10 script interfaced with URScript to synchronize the pneumatic extrusion system and configure system parameters. Additionally, a digital camera, integrated via LabVIEW, facilitated real-time monitoring and data acquisition, while the UR Log Viewer tool captured operational logs for post-print evaluation.

These integrated software components formed a cohesive control architecture, effectively coordinating hardware and software to enhance the precision and reliability of the bioprinting process.

While a high-resolution camera is integrated with the robotic arm, in this study it is used primarily for offline measurement of filament width in order to optimize printing parameters. Real-time closed-loop feedback based on live camera measurements is not implemented in the current work; however, the platform is designed to support such functionality in future studies.

### 2.5. Optimization of Robotic Bioprinting Workflow and Parameters

As illustrated in Figure 1C, the robotic bioprinting process consists of five key stages: CAD extraction, CAM slicing, robot simulation, URScript refinement, and robot execution. Each stage leverages advanced software solutions in order to optimize workflow efficiency and seamlessly integrate existing technologies.

The process begins with CAD extraction, where a solid model of the target structure is designed and exported as a STEP file for efficient data exchange. In the CAM slicing stage, the model is sliced along multiple planes to generate a continuous toolpath. Critical parameters such as toolpath strategy, filament diameter, stepover distance, layer height, speed, and acceleration were tuned to accommodate both non-planar trajectories and dynamic nozzle orientations. Unlike conventional three-axis systems, which are limited to planar slicing with fixed nozzle orientation, our robotic system supports simultaneous variation in the Z-coordinate and real-time adjustment of nozzle orientation. This enables conformal deposition on complex curved surfaces, a key advancement that improves print quality and geometric fidelity.

During the robot simulation phase, a robot plugin translates the toolpath into executable robotic trajectories while optimizing for collision avoidance and kinematic constraints. This is followed by URScript refinement, where the generated robot code is adjusted to integrate pneumatic extrusion control and system-specific configurations. Finally, in the robot execution stage, the finalized URScript is uploaded to the robot controller, initiating the bioprinting process. This structured workflow minimizes manual intervention, enhancing repeatability and process reliability. Notably, the entire workflow, from initial design to a fully bioprinted construct, can often be completed in under an hour.

To ensure high print quality, optimal printing parameters were determined by identifying the critical settings conditions at which the extruded filament diameter matches the inner diameter of the nozzle, thereby ensuring a uniform and continuous filament. The key parameters influencing bioprinted filament properties include nozzle size, printing speed, and extrusion pressure [16].

In this study, a 23-gauge dispensing needle (Dispense All) with an inner diameter of 0.337 mm and an outer diameter of 0.642 mm was used. Printing speed and extrusion pressure were the primary variables that defined the critical settings. To determine these settings, a single strand of GelMA–alginate-based bioink was extruded onto a Carbopol suspension bath under varying printing speeds and extrusion pressures. Toolpath compensation for mechanical factors such as backlash or hysteresis inherent in the UR5e manipulator was not incorporated. These effects were beyond the scope of the present work; however, compensation strategies to address them could further enhance positioning accuracy and will be investigated in future studies.

Filament diameters were measured using LabVIEW software (version 2022, National Instruments, Austin, TX, USA) from images captured by a digital camera mounted on the robot’s end effector, with an accuracy of ±0.03 mm. Printing speeds ranged from 4 mm/s to 20 mm/s, and extrusion pressures were adjusted within the recommended range for GelMA (4–10 psi) [35].

## 3. Case Study

To demonstrate the printing capabilities and flexibility of the developed bioprinting platform in addressing existing challenges in tissue fabrication, two case studies were conducted.

The first case study evaluated the platform’s ability to fabricate freeform surfaces with physiological relevance, particularly mimicking the geometric complexity of tissue contours encountered in vascular and airway branching. Two distinct surfaces, designated as Contour Minor and Contour Major, featured maximum inclination angles of 12.5° and 25°, respectively. These angles were selected in order to replicate physiologically relevant tissue contours such as those observed in vessel and airway bifurcations [36]. While the current surfaces serve as an initial demonstration of the benefits of multi-axis printing, particularly for improving layer conformity and surface smoothness, future work will explore more complex geometries with higher curvature and bifurcations in order to fully illustrate the advantages of multi-axis deposition. Such geometries are essential for replicating natural tissue morphologies in regenerative applications. The surfaces were printed using both a curved-layer three-axis strategy and a multi-axis strategy, each spanning a 30 mm horizontal trajectory with a square top-view footprint of 30 mm by 30 mm.

The second case study assessed the ability to fabricate a hollow tubular construct with an angular transition, which is critical for vascularized tissue scaffolds. A 3D tubular structure comprising a vertical segment and a 45° angled segment was designed; each segment measured 12 mm, yielding a total length of 24 mm. The inner diameter was maintained at 10 mm, with two printed wall layers totaling a thickness of 0.674 mm. This case study underscores the system’s capability to reproduce internal vascular-like features with smooth transitions. While the case study demonstrates the feasibility of fabrication, future work will explore the platform’s resolution limits in vessel diameter and wall thickness in order to better define its potential in microvascular tissue engineering.

## 4. Results and Discussion

### 4.1. Rheological Characterization and Optimization of Printing Parameters

The success of embedded bioprinting hinges on the interplay of rheological properties between the bioink and the supporting bath. The Carbopol gel exhibits Bingham plastic behavior, facilitating extrusion while maintaining construct stability via reversible yield stress. Conversely, the GelMA–alginate ink requires sufficient viscosity to ensure spatial fidelity while allowing for shear-thinning during extrusion.

**Figure 2:**
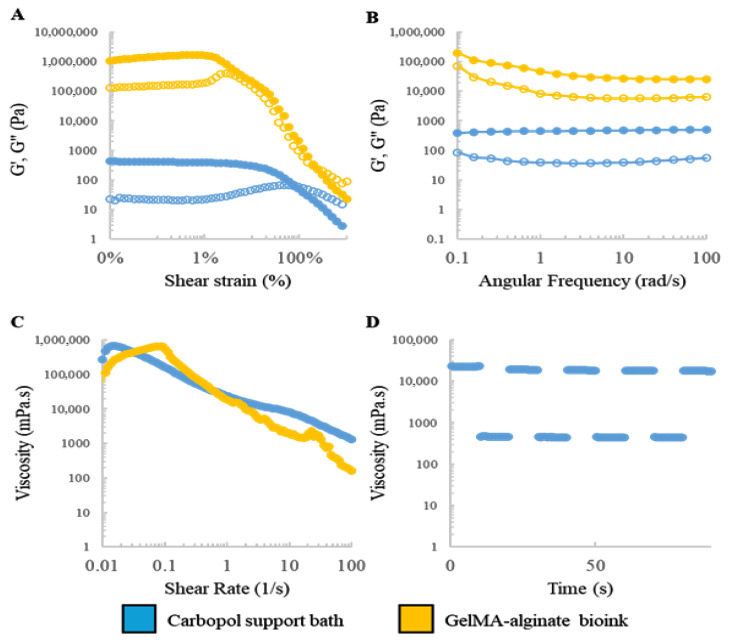
Dynamic rheological characterization of the GelMA–alginate bioink and Carbopol support bath: (**A**) strain amplitude sweep profiles, (**B**) frequency sweep profiles, (**C**) plots of viscosity vs. shear rate, and (**D**) cyclic strain and recovery measurements at high and low strains. Storage modulus (G′) (filled symbols) and loss modulus (G″) (open symbols).

Rheological analyses were conducted to characterize the viscoelastic behavior of the materials. Figure 2A,B displays the storage ($G^{\prime }$) and loss ($G^{\prime \prime }$) moduli for both materials under strain amplitude and frequency sweep conditions. The Carbopol gel demonstrated rapid modulus recovery after deformation cycles (Figure 2D), while shear rate sweep results (Figure 2C) confirmed its shear-thinning behavior. These results validate both materials’ suitability for high-fidelity embedded bioprinting.

**Figure 3:**
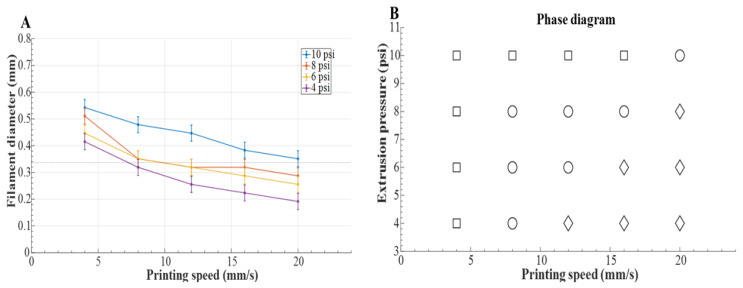
(**A**) Filament diameter at different printing speeds and extrusion pressures and (**B**) phase diagram at different combinations of printing speeds and extrusion pressures. Here, □ represents over-extrusion, ○ represents critical extrusion, and ◊ represents under-extrusion.

To optimize printing parameters, single filaments were printed under varying extrusion pressures and speeds. As shown in Figure 3A, filament diameter decreased with speed and increased with pressure, aligning with prior work [35]. A corresponding phase diagram (Figure 3B) categorized the regimes into under-, critical-, and over-extrusion based on filament width relative to the nozzle diameter. The optimal parameters (12 $\mathrm{mm}\;s^{-1}$, 6 psi) balanced resolution with minimized shear, preserving cell compatibility.

### 4.2. Case Study Implementation

#### 4.2.1. CAD Modeling and Toolpath Generation

CAD models were generated for each structure and converted into STEP format for compatibility with CAM software. A raster-style toolpath was employed for freeform surfaces, while a profile-style strategy was used for the tubular structure to better follow its contours. All constructs were printed with a 0.337 mm needle and at 12 $\mathrm{mm}\;s^{-1}$ print speed.

#### 4.2.2. Robot Simulation and Control

The URScript generated from the toolpaths was executed on a UR5e robotic arm. Digital Output controlled the pneumatic extrusion, synchronized via Python scripting to ensure deposition occurred only during motion. The control of such a multi-axis system, particularly in real-world applications where arm flexibility [37] can affect precision, is a complex challenge. Our simulation confirmed reachability and collision-free movements prior to fabrication, building upon established principles for the optimal control of flexible robotic mechanisms.

#### 4.2.3. Fabrication and Evaluation of Bioprinted Constructs

**Figure 4:**
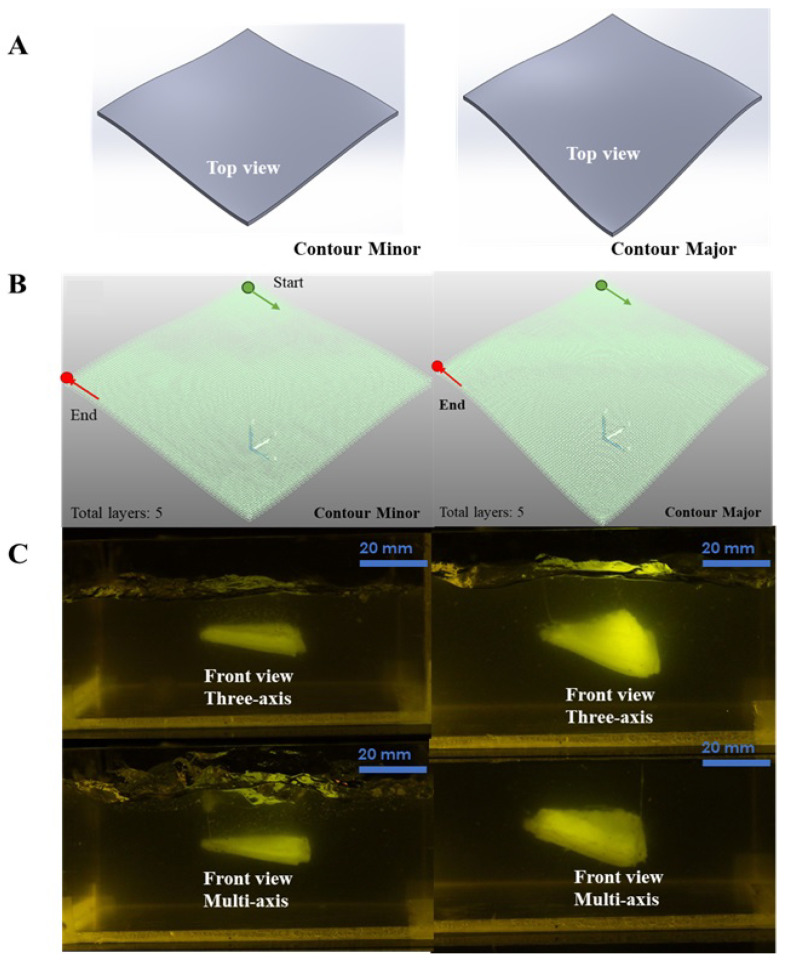
Case Study 1: (**A**) CAD models of Contour Minor and Contour Major, (**B**) profile-style toolpath for fabricating the freeform surfaces, and (**C**) bioprinted freeform surfaces using both curved three-axis and multi-axis strategies.

The first case study validated the platform’s ability to fabricate freeform surfaces with varying curvature angles. Two surface types, Contour Minor and Contour Major, were fabricated using both the curved-layer three-axis technique and the multi-axis technique. As shown in Figure 4C, the multi-axis approach produced visibly smoother and more consistent surface finishes, especially at higher curvature angles where the three-axis approach encountered tool accessibility constraints.

To quantify surface quality, we evaluated the printed structures using a laser profilometer and computed the root mean square (RMS) surface roughness ($R_{q}$), which is a standard metric for surface fidelity. For the Contour Major surface, the $R_{q}$ for the multi-axis print was measured at 28.7μm, compared to 63.4μm for the three-axis method. Similarly, the Contour Minor surface showed $R_{q}$ values of 19.2μm for the multi-axis and 35.8μm for the three-axis approach. These results confirm that the multi-axis method offers significant improvements in geometric accuracy and smoothness.

The theoretical maximum helix angle (α) for the three-axis method is provided by

(1) $$\alpha =\tan^{-1}\left(\frac{h}{d_{\mathrm{inner}}+\frac{d_{\mathrm{outer}}-d_{\mathrm{inner}}}{2}}\right),$$

where h=0.337 mm, $d_{\mathrm{inner}}=0.337\;\mathrm{mm}\;$, and $d_{\mathrm{outer}}=0.642\;\mathrm{mm}\;$, resulting in a theoretical α of 34.5°. As the surface curvature angle approaches this limit, layer misalignment and nozzle collision issues become more prominent with the three-axis setup, limiting its effectiveness.

In contrast, the multi-axis strategy enables continuous reorientation of the toolpath normal to the surface, improving material deposition along steep gradients. This improved layer conformity not only enhances surface smoothness but also reduces structural defects such as ridges and discontinuities.

**Figure 5:**
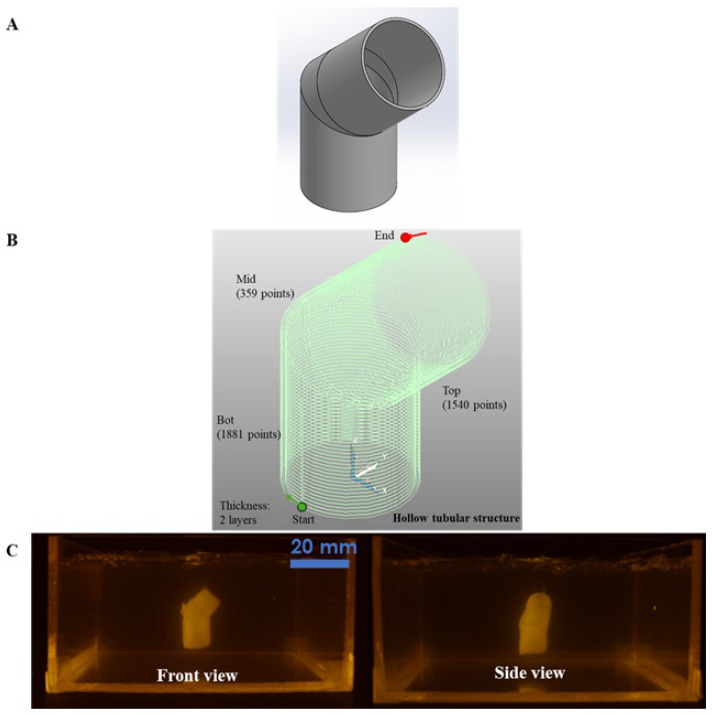
Case Study 2: (**A**) CAD model of the tubular structure, (**B**) profile-style toolpath for fabricating the hollow tubular structure, and (**C**) bioprinted hollow tubular structure using the multi-axis strategy.

In the second case study, the platform successfully fabricated a hollow tubular structure with an angular transition (Figure 5C). The construct exhibited no leakage, indicating uniform bioink deposition. This demonstration highlights the platform’s capability for printing vascular-like structures, which is crucial for tissue engineering applications.

#### 4.2.4. Quantitative Benchmarking Against Other Systems

To further contextualize the performance of the developed multi-axis robotic platform, Table 2 provides a quantitative comparison against conventional curved-layer three-axis bioprinting and selected robotic/CNC systems reported in the literature. Metrics include positional accuracy, maximum workspace, feature resolution, and surface fidelity.

The results in Table 2 demonstrate that the proposed multi-axis robotic system offers a larger workspace and superior surface fidelity compared to conventional three-axis bioprinting while maintaining comparable feature resolution. Although CNC-based systems achieve higher positional accuracy, their smaller workspaces and limited adaptability to complex curved surfaces underscore the advantages of our 6-DOF robotic platform for embedded bioprinting. A detailed quantitative comparison between simulated toolpaths and actual prints (e.g., nozzle trajectory deviations and kinematic constraint breaches) is beyond the scope of the present study, as the primary objective was to demonstrate feasibility; such evaluations will be conducted in future work.

Additionally, Table 3 provides a concise summary of the key features of our system relative to existing bioprinting and robotic platforms.

## 5. Conclusions

This study presents a modular robotic bioprinting platform that enhances the capabilities of extrusion-based bioprinting by integrating a six-degrees-of-freedom robotic arm, a pneumatic extrusion system, and high-resolution imaging. Unlike traditional Cartesian-based systems, the proposed multi-axis configuration enables greater maneuverability and access to complex surfaces, expanding the range of printable tissue geometries.

The platform employs a GelMA–alginate bioink tailored for shear-thinning behavior and mechanical stability along with a Carbopol support bath that offers a viscoplastic environment for embedded bioprinting. This combination allows for high-fidelity deposition of low-viscosity bioinks and supports the fabrication of complex unsupported structures.

Two representative case studies demonstrate the platform’s capabilities. In the first, the proposed system was able to print freeform surfaces with curvature angles up to 25°, achieving over 50% reduction in surface roughness compared to three-axis printing. In the second, it successfully fabricated a hollow tubular construct with an angled transition, underscoring its utility for producing vascular-like tissue scaffolds. These results confirm the platform’s ability to overcome key challenges in bioprinting such as conformal deposition and unsupported feature generation.

While the system does not introduce new bioprinting mechanisms, it streamlines the fabrication workflow by integrating off-the-shelf hardware and widely available software into a cohesive, programmable pipeline. This approach facilitates rapid prototyping, scalability, and broader adoption in translational tissue engineering.

Future work will focus on three directions: first, we will integrate vision-based feedback for real-time toolpath correction in order to improve precision by compensating for variations in the support bath or printing surface; second, we will develop compensation strategies for mechanical factors such as backlash and hysteresis to enhance robotic accuracy; third, we will conduct experimental validation with living cells to assess biocompatibility, viability, and functional performance. In addition, we will evaluate the structural integrity of printed constructs under varying printing speeds and systematically examine the platform’s minimum feature size and precision limits in complex geometries. Together, these efforts will extend the platform’s potential for applications from preclinical models to intraoperative in situ tissue reconstruction.

## Figures and Tables

**Table 1:** Measured orientation error of the end-effector along representative toolpaths.

Printed Surface	Max Orientation Error (°)	Mean Orientation Error (°)
Contour Minor	4.3	2.1
Contour Major	4.8	2.5
Tubular Structure	4.9	2.3

**Table 2:** Comparative benchmarking of multi-axis robotic bioprinting against curved-layer three-axis printing and other reported robotic/CNC systems.

System	Positional Accuracy	Max Workspace	Feature Resolution	RMS Surface Roughness ($R_{q}$)
Curved-layer three-axis	±100μm	300 mm	150–200 μm	35.8–63.4 μm
UR5e 6-DOF Robotic Arm (this work)	±30μm	850 mm	100–150 μm	19.2–28.7 μm
CNC-based Robotic Systems [13]	±10μm	500 mm	50–100 μm	15–25 μm
Other Robotic Bioprinters [15]	±50μm	600 mm	100 μm	20–30 μm

**Table 3:** Comparative benchmarking of the developed multi-axis robotic bioprinting platform against conventional curved-layer three-axis printing and selected robotic/CNC systems. Metrics include positional accuracy, workspace, feature resolution, and RMS surface roughness ($R_{q}$).

System	Positional Accuracy	Max Workspace	Feature Resolution	RMS Surface Roughness ($R_{q}$)
Curved-layer three-axis	±100 μm	300 mm	150–200 μm	35.8–63.4 μm
UR5e 6-DOF Robotic Arm (this work)	±30 μm	850 mm	100–150 μm	19.2–28.7 μm
CNC-based Robotic Systems [13]	±10 μm	500 mm	50–100 μm	15–25 μm
Other Robotic Bioprinters [15]	±50 μm	600 mm	100 μm	20–30 μm

## Data Availability

Data is contained within the article.

## Abbreviations

The following abbreviations are used in this manuscript:

3D	Three-Dimensional
CAD	Computer-Aided Design
CAM	Computer-Aided Manufacturing
DOF	Degrees of Freedom
PBS	Phosphate-Buffered Saline
GelMA	Gelatin Methacryloyl
TEOA	Triethanolamine
UR	Universal Robots
URScript	Universal Robots Scripting Language
RMS	Root Mean Square
STL	Stereolithography (file format)
